# Proposal of Shuttleworthella gen. nov. and Nostocoides gen. nov. as replacement names for the illegitimate prokaryotic generic names Shuttleworthia and Tetrasphaera, respectively

**DOI:** 10.1099/ijsem.0.006318

**Published:** 2024-04-04

**Authors:** Umakant Bhoopati Deshmukh, Aharon Oren

**Affiliations:** 1 Institution of Higher Learning, Research and Specialized Studies Centre, Department of Botany, Janata Mahavidyalaya, Chandrapur 442 401, Maharashtra, India; 2 Department of Plant and Environmental Sciences, The Institute of Life Sciences, The Hebrew University of Jerusalem, Edmond J. Safra Campus, Jerusalem 9190401, Israel

**Keywords:** illegitimate names, *Nostocoides*, *Shuttleworthella*, *Shuttleworthia*, *Tetrasphaera*

## Abstract

The prokaryotic generic name *Shuttleworthia* Downes *et al*. 2002 is illegitimate because it is a later homonym of the plant genus *Shuttleworthia* Meisner 1840 and the mollusk genus *Shuttleworthia* Baker 1941 (Principle 2 and Rule 51b(5) of the International Code of Nomenclature of Prokaryotes). We therefore propose the replacement generic name *Shuttleworthella*, with type species *Shuttleworthella satelles* comb. nov. The prokaryotic generic name *Tetrasphaera* Maszenan *et al*. 2000 is illegitimate because it is a later homonym of *Tetrasphaera* Popofsky 1913 (Protozoa, Radiolaria) and of *Tetrasphaera* Górka 1965 (a fossil dinoflagellate) (Rule 51b(4) of the International Code of Nomenclature of Prokaryotes). We therefore propose the replacement generic name *Nostocoides*, with type species *Nostocoides japonicum* comb. nov.

According to Principle 2 and Rules 51b(5) of the International Code of Nomenclature of Prokaryotes (ICNP) [1], a name or combination validly published on or after 1 January 2001 is illegitimate when it is a later homonym of a name or combination validly published or available under the International Code of Nomenclature for algae, fungi, and plants or the International Code of Zoological Nomenclature. According to Rule 51b(4), a name or combination validly published before 31 December 2000 is illegitimate when it is a later homonym of a name of a taxon of prokaryotes, fungi, algae, protozoa or viruses. Based on these Rules, we identified two illegitimate prokaryote genus names, *Shuttleworthia* Downes *et al*. 2002 and *Tetrasphaera* Maszenan *et al*. 2000. We here propose replacement names for these genera.

## Illegitimacy of the name *Shuttleworthia* Downes *et al*. 2002

The prokaryotic genus *Shuttleworthia* (*Lachnospiraceae, Lachnospirales, Clostridia*, *Bacillota*) was described by Downes *et al*. in 2002 [2]. The type species and thus far only species is *Shuttleworthia satelles*. The generic name *Shuttleworthia* is a later homonym of *Shuttleworthia* Meissner 1840 (*Tracheophyta*, *Magnoliopsida*, *Lamiales*, *Verbenaceae*) [3] and of *Shuttleworthia* Baker 1941 (Animalia, Mollusca) [4]. Based on Principle 2 and Rule 51b(5) of the ICNP, the name *Shuttleworthia* proposed in 2002 for a prokaryotic genus is therefore illegitimate. In accordance with Rule 54, we here propose a replacement name, conserving the meaning of the illegitimate name as originally published.

## Description of *Shuttleworthella* Deshmukh and Oren gen. nov.

*Shuttleworthella* (Shut.tle.worth.el’la. N.L. fem. n. *Shuttleworthella*, named after the British microbiologist Cyril Shuttleworth).

Earlier illegitimate homotypic synonym: *Shuttleworthia* Downes *et al*. 2002.

The description of the genus is as given for *Shuttleworthia* [2].

The type species is *Shuttleworthella satelles* (Downes *et al*. 2002) Deshmukh and Oren.

## Description of *Shuttleworthella satelles* (Downes *et al*. 2002) Deshmukh and Oren comb. nov.

*Shuttleworthella satelles* (sa.tel’les. L. masc. n. *satelles*, a satellite or attendant upon a distinguished person, referring to the satelliting appearance of older cultures).

Basonym: *Shuttleworthia satelles* Downes *et al*. 2002.

The description of the species is as given for *Shuttleworthia satelles* [2].

The type strain is VPI D143K-13^T^ (=CCUG 45864^T^=DSM 14600^T^), isolated from human subgingival plaque and periodontal pockets in patients with periodontitis. The GenBank/EMBL/DDBJ accession number for the 16S rRNA gene is AF399956.

## Illegitimacy of the name *Tetrasphaera* Maszenan *et al.* 2000

The prokaryotic genus *Tetrasphaera* (*Intrasporangiaceae, Micrococcales, Actinomycetes*, *Actinomycetota*) was described by Maszenan *et al*. in 2000 [5]. The type species is *Tetrasphaera japonica*. The genus currently contains eight species with validly published names. The generic name *Tetrasphaera* is a later homonym of *Tetrasphaera* Popofsky 1913 (Protozoa, Radiolaria) [6] and of *Tetrasphaera* Górka 1965 (a fossil dinoflagellate) [7]. It is also a homonym of *Tetrasphaera* Timofeev and Herman 1979 (fossil Acritarcha, a group of uncertain taxonomic affiliation) [8]. Based on Rule 51b(4) of the ICNP, the name *Tetrasphaera* proposed in 2000 for a prokaryotic genus is therefore illegitimate. In accordance with Rule 54, we here propose a replacement name, conserving the meaning of the illegitimate name as originally published. We here reclassify five *Tetrasphaera* species as new combinations. We did not reclassify *Tetrasphaera duodecadis* (Lochhead 1958) Ishikawa and Yokota 2006, *Tetrasphaera elongata* Hanada *et al*. 2002 and *Tetrasphaera remsis* Osman *et al*. 2007, as they were later reclassified as *Phycicoccus duodecadis* (Lochhead 1958) Nouioui *et al*. 2018, *Phycicoccus elongatus* (Hanada *et al*. 2002) Nouioui *et al*. 2018, and *Knoellia remsis* (Osman *et al*. 2007) Nouioui *et al*. 2018, respectively.

## Description of *Nostocoides* Deshmukh and Oren gen. nov.

*Nostocoides* (Nos.toc.o′i.des. N.L. neut. n. *Nostoc*, a cyanobacterial genus; L. suff. –*oides* (from Gr. suff. –*eides*, that which is seen, form, shape, figure), resembling; N.L. neut. n. *Nostocoides*, resembling *Nostoc*).

Earlier illegitimate homotypic synonym: *Tetrasphaera* Maszenan *et al*. 2000. Heterotypic synonyms: ‘*Candidatus* Nostocoida’ Blackall *et al*. 2000 [9,10]; ‘*Candidatus* Nostocoides’ corrig. Blackall *et al*. 2000 [11].

The description of the genus is as given for *Tetrasphaera* [5] as emended by Ishikawa and Yokota [12].

The type species is *Nostocoides japonicum* (Maszenan *et al*. 2000) Deshmukh and Oren.

## Description of *Nostocoides australiense* (Maszenan *et al.* 2000) Deshmukh and Oren comb. nov.

*Allotetrasphaera australiense* (aus.tra.li.en’se. N.L. neut. adj. *australiense*, of Australia, from where the isolates originated).

Basonym: *Tetrasphaera australiensis* Maszenan *et al*. 2000.

The description of the species is as given for *Tetrasphaera australiensis* [5].

The type strain is Ben 109^T^ (=JCM 21391^T^=KCTC 39584^T^=NBRC 103087^T^), isolated from activated sludge biomass in Australia. The GenBank/EMBL/DDBJ accession number for the 16S rRNA gene is AF125091.

## Description of *Nostocoides japonicum* (Maszenan *et al.* 2000) Deshmukh and Oren comb. nov.

*Nostocoides japonicum* (ja.po’ni.cum. N.L. neut. adj. *japonicum*, pertaining to Japan).

Basonym: *Tetrasphaera japonica* Maszenan *et al*. 2000.

The description of the species is as given for *Tetrasphaera japonica* [5].

The type strain is T1-X7^T^ (=DSM 13192^T^=JCM 21381^T^=NBRC 103088^T^), isolated from activated sludge in Japan. The GenBank/EMBL/DDBJ accession numbers for the 16S rRNA gene and the whole genome sequence are AF125092 and GCA_001046855.

## Description of *Nostocoides jenkinsii* (Mckenzie *et al.* 2006) Deshmukh and Oren comb. nov.

*Nostocoides jenkinsii* (jen.kin’si.i. N.L. gen. n. *jenkinsii*, of Jenkins, referring to David Jenkins, an American environmental engineer, who has made a considerable contribution to our understanding of the filamentous bacteria causing bulking and foaming in activated sludge processes).

Basonym: *Tetrasphaera jenkinsii* McKenzie *et al*. 2006 emend. Nouioui *et al*. 2018.

The description of the species is as given for *Tetrasphaera jenkinsii* [10,13].

The type strain is Ben 74^T^ (=DSM 17519^T^=JCM 15590^T^=NCIMB 14128^T^), isolated from activated sludge systems in Australia. The GenBank/EMBL/DDBJ accession numbers for the 16S rRNA gene and the whole genome sequence are DQ007321 and GCA_001046875.

## Description of *Nostocoides vanveenii* (Mcenzie *et al.* 2006) Deshmukh and Oren comb. nov.

*Nostocoides vanveenii* (van.vee’ni.i. N.L. gen. n. *vanveenii*, of van Veen, referring to the late Dutch microbiologist W. L. van Veen, who originally isolated this filamentous bacterium from activated sludge)

Basonym: *Tetrasphaera vanveenii* McKenzie *et al*. 2006.

The description of the species is as given for *Tetrasphaera vanveenii* [10].

The type strain is Ben 70^T^ (=DSM 17518^T^=JCM 15591^T^=KCTC 39576^T^=NCIMB 14127^T^), isolated from an activated sludge plant in Australia. The GenBank/EMBL/DDBJ accession number for the 16S rRNA gene is DQ007320.

## Description of *Nostocoides veronense* (McKenzie *et al.* 2006) Deshmukh and Oren comb. nov.

*Nostocoides veronense* (ve.ro.nen’se. N.L. neut. adj. *veronense*, of Verona, Italy, from where the first isolates originated).

Basonym: *Tetrasphaera veronensis* McKenzie *et al*. 2006.

The type strain of *Tetrasphaera veronensis* McKenzie *et al*. 2006 was previously known as ‘*Candidatus* Nostocoida limicola’ Blackall *et al*. 2000 [9,10] and ‘*Candidatus* Nostocoides limicola’ corrig. [11].

The description of the species is as given for *Tetrasphaera veronensis* [10].

The type strain is Ver 1^T^ (=DSM 17520^T^=JCM 15592^T^=KCTC 39577^T^=NCIMB 14129^T^), isolated from an activated sludge plant in Verona, Italy. The GenBank/EMBL/DDBJ accession number for the 16S rRNA gene is Y14595.

## Abbreviation

ICNP: International Code of Nomenclature of Prokaryotes
